# The immunomodulatory role of carbon monoxide during transplantation

**DOI:** 10.1186/2045-9912-3-1

**Published:** 2013-01-07

**Authors:** Mariane Tami Amano, Niels Olsen Saraiva Camara

**Affiliations:** 1 Laboratory of Transplantation Immunobiology, Department of Immunology, Institute of Biomedical Sciences, University of Sao Paulo, Sao Paulo, Brazil

**Keywords:** CO, HO-1, Immune response, Transplant

## Abstract

The number of organ and tissue transplants has increased worldwide in recent decades. However, graft rejection, infections due to the use of immunosuppressive drugs and a shortage of graft donors remain major concerns. Carbon monoxide (CO) had long been regarded solely as a poisonous gas. Ultimately, physiological studies unveiled the endogenous production of CO, particularly by the heme oxygenase (HO)-1 enzyme, recognizing CO as a beneficial gas when used at therapeutic doses. The protective properties of CO led researchers to develop uses for it, resulting in devices and molecules that can deliver CO *in vitro* and *in vivo*. The resulting interest in clinical investigations was immediate. Studies regarding the CO/HO-1 modulation of immune responses and their effects on various immune disorders gave rise to transplantation research, where CO was shown to be essential in the protection against organ rejection in animal models. This review provides a perspective of how CO modulates the immune system to improve transplantation and suggests its use as a therapy in the field.

## Review

### Transplantation

End-stage organ failure often requires transplantation, and the number of solid organ transplants reached 106,900 worldwide in 2010 according to the Global Observatory on Donation and Transplantation (http://www.transplant-observatory.org). One of the most common solid organ transplants is the kidney. Even for renal diseases that can be treated with various therapies, transplants increase the quality of life in most cases and are a financially attractive solution. More than 73,000 kidney transplants were performed in 2010; in comparison, approximately 21,000 liver transplants were performed (http://www.transplant-observatory.org). Although there are a high number of solid organ transplants, graft loss following chronic allograft dysfunction is still a major concern during transplantation [1,2]. For cases in which chronic rejection does not occur, side-effects due to the use of immunosuppressants are the main cause of mortality [3]. A third concern is the shortage of organs that has forced the donor pool to include extended criteria and non-heart beating donors, which are more susceptible to delayed graft function (DGF) [4]. All of these complications reinforce the search for new transplantation therapies.

### Immune system in ischemia and reperfusion

The immune system is divided into the innate and adaptive immune responses. The innate immune response is known as the first line of defense, and it depends mostly on inflammatory components. It is faster and less specific than the adaptive response. In contrast, the adaptive response involves the participation of lymphocytes, and it generates memory. It takes longer to build an adaptive response, but such responses are more specific than innate responses. While adaptive immune responses are an excellent system for fighting pathogens, they are also very effective against allograft acceptance. In solid organ transplantation, the graft is subjected to ischemia prior to being transplanted. Ischemia and reperfusion (IR) is the first step in which the immune system acts to avoid the survival of the graft. Ischemia is defined as the cessation of arterial blood flow, which leads to oxygen deprivation of the cells. Cold ischemia is most often used in transplantation, whereby the organ is harvested and kept in a cold solution. There is also warm ischemia, which involves the blockade of blood flow by trauma, such as during a stroke [5]. IR causes cell damage [6,7], and it is associated with DGF and primary graft nonfunction [8,9].

Microvascular damage initiates inflammation by upregulating complement [10], Toll-like receptors (TLRs) [5], TLR ligands [9], and leukocyte adhesion molecules [11].

The complement system is a cascade of proteins that participates in the inflammatory response and produces the membrane attack complex (MAC). C5a, one of the products of complement activation, is involved in IR injury by attracting and stimulating the degranulation of neutrophils, as well as upregulating CXC-motif chemokines [12]. Blockade of the C5a receptor during cold ischemia impairs IR damage by diminishing tubular cell apoptosis [13]. MAC formation can lead to cell lysis, but it can also activate tubular epithelial cells [14-16] to upregulate proinflammatory and fibrotic factors, such as IL-6, TNF, ICAM-1 and collagen [14-17]. The complement inhibitor decay-accelerating factor (DAF) was shown to be absent in mice that are more susceptible to MAC-induced microvascular injury following IR [18]. The deposition of MBL, C3, C6 and C9 in the kidney following IR [19] and the deficiency of Crry (a C3 inhibitor) increased the susceptibility of mice to kidney IR injury [20], corroborating the notion that complement activation during IR contributes to the inflammatory response.

TLRs are a component of the innate immune response because they recognize pathogen- and damage-associated molecular patterns, and they have been implicated in several inflammatory diseases. The absence of TLR4 and/or TLR2 protects mice from IR injury, improving cardiac function [21,22]. TLR2 expression was increased in the liver following IR, and it was associated with higher levels of TNF [23]. However, the lack of TLR2 was not able to protect animals from liver IR injury, while TLR4-deficient animals were protected. This protection was associated with reduced levels of TNF, and it was shown to be dependent on intrahepatic HO-1 expression [24]. TLR2- and MyD88 (adapter protein for most TLRs)-deficient mice displayed decreased tubular epithelial apoptosis, cellular infiltration and dysfunction [25,26]. TLR4^−/−^ animals were also protected from IR with improved renal function, diminished chemokine production and fewer cellular infiltrates [27,28]. The increase in TLR4 following IR was accompanied by an upregulation of HMGB-1, hyaluronan and brevican [27], which suggested that these ligands could be responsible for the downstream activation of TLRs, thereby improving the inflammatory response and contributing to IR injury.

Leukocyte adhesion molecules are often associated with cell migration during inflammatory responses. There are three main groups of leukocyte adhesion molecules: integrins (VLA-4, CD11/CD18) [29,30], immunoglobulin super family members (ICAM-1, VCAM-1, CD4, CD8) [31] and selectins (E, P, L-selectin) [32]. In animal models, the administration of monoclonal antibodies against leukocyte adhesion molecules was able to attenuate IR injury in many organs, including the heart, liver and skeletal muscle [33]. The administration of anti-CD11a and anti-CD11b monoclonal antibodies prior to renal ischemia prevented renal injury with lower serum creatinine levels, but it did not abolish neutrophil migration [34]. IR upregulates ICAM-1 expression in the murine kidney, and the absence of this molecule protects animals from IR injury [35]. Although the first two adhesion molecule groups seem to be involved at least partially in IR damage, the selectin group is minimally involved. L-selectin deficient mice presented similar levels of neutrophil infiltration and renal function when compared to wild type controls [36]. These studies confirmed the role of inflammation during IR injury and led us to question the participation of immune cells in this stage of the transplant process.

As mentioned previously, immune cells infiltrate organs during reperfusion. Neutrophils usually accumulate in the organ following IR in mouse models [35,37], and the depletion of this cell type prevents acute kidney injury (AKI) [35]. It remains unclear how neutrophils migrate and become activated in the ischemic organ, but they seem to be fundamental for IFN-γ and IL-17 production [37,38]. Invariant natural killer T (iNKT) cells were also shown to be important for the control of IFN-γ-producing neutrophils in a renal IR model [37]. iNKT cells are also involved in hepatic IR injury via CD1d activation [39]. In lung IR, these cells are the primary IL-17 producers [40].

Another important innate immune cell is the macrophage. Macrophages are phagocytic, like neutrophils, but they are known as antigen presenting cells (APC) because they present antigens to T cells. These cells were shown to infiltrate organs via CCR2-CX3CR1 upon ischemia, with a slight delay when compared to neutrophils [41]. The depletion of macrophages by liposomal clodronate prior to IR prevented AKI, and the adoptive transfer of these cells reconstituted the injury [42,43]. Neutrophil- and iNKT cell-derived IFN-γ is a potent activator of macrophages, leading to increased production of the proinflammatory cytokines IL-1α, IL-6, TNF and IL-12 [37].

Dendritic cells (DCs) are also APCs and are considered a bridge between innate and adaptive immunity. Blocking the CD80/CD86 costimulatory molecules to prevent T cell activation reduced AKI [44]. Dong *et al.*[45] demonstrated that renal DCs were able to activate T cells from the draining lymph node after IR. In another study [46], they showed that renal DCs displayed elevated expression of activation molecules (CD80, CD86, MHC class II and CD40) following IR, as well as increased expression of IL-6, MCP-1 and RANTES. Furthermore, they established that DCs were the main source of TNF in the kidney after IR. DCs and macrophages are the primary cell types that express TLRs, which suggests that these cells are partially responsible for the involvement of TLRs during IR injury.

B cells have several similarities with DCs and macrophages in that they also are able to process and present antigen to T cells via MHC class II. B cells contribute to IR injury in several models: intestine, heart, kidney and skeletal muscle [47-52]. Furthermore, B cell-deficient mice are protected from renal IR injury [50,52]. Complement receptor (CR) 2 deficient-mice, which are defective in B-1 cells and are therefore immunoglobulin (Ig) M-deficient, are protected from IR muscle injury [51].

The adaptive immune response depends on a series of events, making it a lengthy process. Therefore, T cells, the leading actors of this process, had not been frequently associated with IR injury because it is an immediate response. More recently, a collection of studies has changed this idea and suggested an important role of T cells in IR injury. In a rat model of IR, the use of FTY720 (2-amino-2-[4-octylphenyl]-1,3-propaneldiol hydrochloride), a synthetic analog of sphingosine that blocks T cell circulation from the lymph node to the peripheral blood, improved microcirculation, decreased liver damage and decreased IL-6 and TLR4 expression [53]. In lung IR, CD4^+^ T cells were shown to have a major role in stimulating chemokine production and neutrophil chemotaxis, which in turn contributed to IR injury [54]. Shigematsu *et al.*[55] demonstrated that B cells, CD4^+^ and CD8^+^ T cells are involved in the proinflammatory and prothrombogenic phenotype of intestinal IR. In the kidney, the elimination of CD4^+^ T cells with MHC class II knockout mice or anti-CD4 antibodies led to improved renal function following IR [56]. The depletion of CD4^+^ T cells, but not CD8^+^ T cells, diminished injury after hepatic and renal IR [57,58]. CD4+ T cells were shown to be autoreactive following IR [59], and the transfer of DO11.10 (TCR OVA-specific) CD4+ T cells to nude mice, which are normally protected from renal IR injury, conferred renal damage [60]. These studies strongly suggest the participation of CD4^+^ T cells in IR injury, and this injury seems to be dependent on T cell activation. However, the specificity of this activation remains unclear.

**Figure 1:**
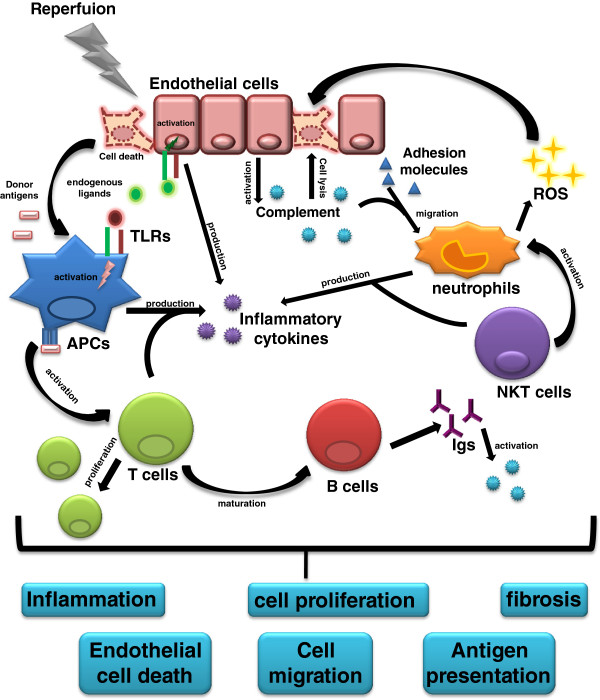
**Immune response activation during reperfusion and transplantation.** Reperfusion can lead endothelial cells to death initiating the immune response. Endogenous ligands are released and recognized by Toll-like receptors (**TLRs**) on antigen presenting cells (**APCs**) or endothelial cells. This activation generates inflammatory cytokines enhancing the inflammatory response and activating other cells from the immune system. During reperfusion, complement proteins can also be activated by the decreased expression of complement inhibitors by endothelial cells. This activation can generate the membrane attack complex leading to endothelial cell lysis. Complement activation can also produce chemokines and anaphylatoxins, and together with an increase in adhesion molecules expression, neutrophils migrate to the graft and produce more inflammatory cytokines and reactive oxygen species (**ROS**), which can contribute to cell death. Natural killer T (**NKT**) cells contribute to neutrophils activation and to cytokines production. During reperfusion, T cells in the lymph node are somehow activated, amplifying cytokines production and leading to B cells maturation, providing immunoglobulins (**Igs**) release. Igs can activate complement and act as opsonins, contributing to the whole process of immune response. This activation persists after transplantation, and donor antigens enhance the immune response when they are processed by APCs (donor or recipient) in the graft that migrate to the lymph node and present them to T cells. T cells can proliferate and amplify the response with an increase in cytokines. The activation of all these components contributes to graft rejection by establishing the local inflammation, leading to endothelial cell death, cell proliferation and cell migration. Donor antigen presentation reinforces the whole process and the persistence of the immune response activation in the graft can change the cytokine profile and favors the fibrosis development.

Altogether, IR is a complex process that involves activation of both the innate and adaptive immune systems, leading to complications in graft acceptance (Figure 1).

### Immune system in transplantation

The general concept of allograft rejection surmises that T cells react to alloantigens presented by donor and/or recipient APCs to trigger cytotoxicity and inflammation. With new advances in transplant research, the involvement of the immune system in this process has changed. Much like IR injury, the transplantation process is now thought to involve both immune responses [61] (Figure 1). During heart or renal rejection, the complement is activated, and it can be detected in the blood or urine [62,63] and in the graft itself [64]. In human kidney allografts, tubular epithelial cells generate complement components and become the primary target of their activation [65]. In a mouse model of kidney transplantation, kidneys from C3^−/−^ donors survived for a long period in a fully mismatched recipient without any immunosuppression, providing evidence of the role of donor-produced C3 in kidney rejection [66]. Human donors with a natural defect in mannose binding lectin (MBL), a protein associated with complement activation, improved the chance of cardiac allograft acceptance [67], whereas heart donor DAF^−/−^ mice accelerated graft rejection [68]. These data suggest two mechanisms for the involvement of complement in allograft rejection: the direct activation of complement in epithelial cells or an indirect role of complement by favoring immune cell activation.

Recipients TLR2^−/−^ and MyD88^−/−^ mice had chronic allograft damage attenuated. These deficiencies also reduced the infiltration of DCs, macrophages and T cells into the graft, leading to decreased expression of IL-6, IL-10, monocyte chemotactic protein-1 (MCP-1) and IL-12. Fibrotic factors were also diminished in these models via decreased collagen types I and III compared to wild type controls [69]. The downregulation of TLR2 and TLR4 by cyclosporine A and Serp-1 co-treatment impaired T cell and macrophage intragraft infiltration and allowed for indefinite graft survival [70]. It was additionally shown that TLR4 is constitutively expressed in donor organs, and TLR4 and HMGB-1 expression are increased in non-heart beating donor kidneys [71,72]. In liver transplantation, TLR2, TLR4, HSP60 and HSP70 were increased during reperfusion, with a peak at 3 h [73]. Patients with acute liver transplant rejection have shown increased CD14+TLR2+ monocytes [74]. TLRs are involved in organ transplantation, and their activation may modulate immune cells that contribute to allograft rejection.

Innate NK cells, which are usually associated with protection against tumors and viral infections, were shown to infiltrate grafts during allogeneic heart transplantation. Associated with this infiltration was the upregulation of their receptor NKG2D as well was their ligands retinoic acid early inducible (RAE-1) and minor histocompatibility antigen H60 [75]. In mouse models, NK cells were shown to be important for the tolerance of islet and skin allografts [76,77]. Together with other studies [61], NK cells appear to participate in the graft progress. However, they appear to promote both tolerance and rejection. Therefore, further investigation is required to understand the relevance of these cells in transplantation models.

Adaptive immunity during transplantation has been extensively studied, and its role in allograft tolerance and rejection is well established. CD4^+^ T cells have long been known to promote allograft rejection [78]. Although CD8^+^ T cells cannot initiate rejection independently, they exert cytotoxic functions via Fas/Fas-L, contributing to the loss of the graft [79]. Valujskikh *et al.*[80] summarized the mechanisms of T cell involvement during transplantation. The classical activation of CD80/CD86 on APCs through T cell CD28 ligation induces cytokine production (IL-2, TNF, IFN-γ) [81], and this is known to lead to allograft rejection. Similarly, CD40/CD154L amplifies T cell activation, which yields the same outcome as costimulation. Other costimulatory molecules seem to function similarly, such as the interaction between ICOS/B7RP-1 and CD134/CD134L. Animal studies have shown that blocking the ICOS/B7RP-1 interaction can prolong allograft survival in heart, liver and islet transplantation models [82-86]. Although the disruption of the CD134/CD134L interaction was not able to provide long-term graft survival on its own, it could when combined with other therapies [87]. Inhibiting the interaction of PD-1/PD-L1 with an anti-PD-L1 antibody had the opposite effect and accelerated skin graft rejection [88]. However, diminishing PD-1 signaling in combination with anti-CD154 delayed islet rejection [89]. Apart from naïve T cell interactions, memory T cells also play a role in allograft rejection. Zhang *et al.* showed that sequestering alloreactive memory CD4^+^ T cells improved graft survival in a heart transplantation model [90], and CD4^+^ T cell subsets were involved. The Th1 subset was thought to be the most important T helper cell in transplantation by promoting the generation of cytotoxic cells, the activation of APCs and antibody production [91,92], while the Th2 subset was seen as a regulatory cell in this model [93]. With the discovery of new T helper cell subsets, including Th17 (produces IL-17 and is associated with inflammatory disorders [94]) and Tregs, this paradigm has been revised: Th17 cells are now recognized as promoting graft rejection along with Th1 cells [93,94]. In humans, IL-23, a cytokine that induces Th17 differentiation, and IL-17 are elevated in the serum from patients who have had hepatic rejection [95]. IL-17 was also increased in the bronchoalveolar lavage of lung transplant patients with acute rejection [96] as well as in the urine of patients with subclinical kidney rejection [97]. Several mouse models have confirmed that IL-17 favors allograft rejection [98,99]. With the discovery of Tregs, the Th2 subset has lost its role as a protector cell in several models, allowing this new regulatory subset to take its place [100,101].

These works summarize the importance of APC-T cell interactions against the foreign graft and how important it is to control their cross-talk following transplantation.

### The classical and new concepts of carbon monoxide

The well-known odorless, colorless and tasteless gas carbon monoxide (CO) was originally described to bind hemoglobin with 140 times greater affinity than oxygen (O_2_) by Haldane in 1895 [102], when it was classified as a cumulative poison. It is currently known that this affinity is approximately 210–250 times greater than O_2_. In 1906, Nasmith and Graham [103] confirmed the poisonous character of CO, showing that this gas prevented O_2_ from reaching tissues. However, they also showed an increase in erythrocytes in the presence of elevated CO levels, similar to those found at high altitudes. This indicated that the body could stand higher levels of CO in certain situations and not succumb to it. The authors did not emphasize this discovery, and CO continued to be popularly associated as a villain for many years.

In 1952, Sjöstrand proved that CO was present in our body and that hemoglobin decomposition could produce CO [104]. Furthermore, increased heme levels were found to increase endogenous CO production [105]. It was only in 1968 that Tenhunen *et al.* showed a connection between heme oxygenase (HO) and CO [106]. They provided evidence that CO and bilirubin were by-products of the HO-mediated cleavage of heme [106,107]. CO was also shown to be produced by other mechanisms, including phenol oxidation [108,109], the hormone progesterone [110] and the peroxidation of microsomal lipids and phospholipids [111-113]. Nevertheless, the majority of CO production in the body is dependent on HO activation [114].

HO is an enzyme that can open the heme ring in the presence of O_2,_ nicotinamide adenine dinucleotide phosphate NADPH and (NADPH)-cytochrome P450 reductase, thus cleaving heme into biliverdin, iron and CO [115,116]. The first isoform of HO-1 was described as inducible in 1974 [117,118], while the other two isoforms (HO-2 and HO-3) were found to be constitutive [119,120]. HO-1 (32 kDa) is localized to microsomes and is induced in mammalian tissues, while HO-2 (36 kDa) is present in mitochondria and is expressed in the brain, testes, endothelium, kidney, liver and gastrointestinal tract [121]. HO-3 was found to be a pseudogene derived from the HO-2 gene [122].

HO-1, also known as heat shock protein 32, has been extensively studied for its protective role. It was shown to have anti-proliferative [123], anti-apoptotic [124], anti-oxidant [125] and anti-inflammatory [126] effects.

HO-2 [127] and HO-1 [128,129] knockout mice broadened our knowledge of HO and its by-products, and they confirmed its anti-inflammatory role through their spontaneous development of an inflammatory phenotype. Two years later, the first case of HO-1 deficiency in humans was described. HO-1-deficient people share similarities with HO-1 knockout mice, as they display tissue iron deposition, lymphadenopathy, leukocytosis and sensitivity to oxidative stress injuries [130].

The discovery of HO-1 as a potential mechanism of immune therapy, and the connection of this enzyme to CO production, raised new ideas about this gas and implicated it as a novel therapy.

After a long absence of CO studies, physiological studies demonstrated that CO was a neurotransmitter in 1993 [131]. With this new vision and with the discoveries of other gases [132], CO began to be investigated as a potential therapy.

Studies concerning the mechanism of action of CO have shown that it binds to the heme moiety of soluble guanylyl cyclase (sGC), leading to cyclic guanosine monophosphate (cGMP) activation [133,134]. CO-induced cGMP is involved in vascular relaxation [133,134], the inhibition of vascular smooth cell proliferation [135,136], the inhibition of platelet aggregation [137] and anti-apoptotic action on pancreatic β cells [138]. The anti-apoptotic properties of CO have been extensively studied because of their possible indication of CO as a therapeutic agent for several disorders. CO was shown to prevent mitochondrial permeabilization, inhibiting the intrinsic apoptotic pathway [139]. In macrophage lineages, CO inhibited cytochrome c oxidase and the generation of mitochondrial ROS [140]. In astrocytes, CO was shown to induce cytochrome c oxidase activity and increased Bcl-2 expression, which rapidly interacted with cytochrome c oxidase to prevent apoptosis [141]. Endothelial cells require activation of the p38/mitogen-activated protein kinase (MAPK) pathway by CO to prevent TNF-induced cell death [142]. In contrast, CO promotes Fas/CD95-induced cell death by inhibiting activation of the ERK/MAPK pathway in T cells [143].

Although CO activates cGMP, nitric oxide (NO) activates it more potently [144]. The relationship between these two molecules seems to involve a complex negative feedback loop: NO induces HO-1 expression and consequently CO production [145], while conversely, HO-1 and CO inhibit NO synthesis activity [146,147].

Several groups began developing ways to release CO in order to manipulate the quantity of gas. CO at 250 ppm was shown to induce macrophage phagocytosis, and the same condition was described to be beneficial in many animal disease models [148]. In 2002, Chauveau *et al.*[149] used methylene chloride as a pro-drug to induce CO release by hepatic enzyme catabolism. Because methylene chloride use is dependent on the condition of the liver, Motterlini *et al.* searched for new CO-releasing molecule (CORM) candidates [150]. They identified molecules based on heavy metals surrounded by carbonyl groups, such as iron pentacarbonyl [Fe(CO)5], dimanganese decacarbonyl [Mn2(CO)10] and tricarbonyldichlororuthenium (II) dimers [Ru(CO)3Cl2]2. All of the compounds could convert deoxymyoglobin to carbonmonoxymyoglobin, which indicates that CO has been released from the metal complexes. These molecules were able to attenuate coronary vasoconstriction *ex vivo* and reduce acute hypertension *in vivo*. The same results were observed after hemin treatment, which stimulates CO release through HO-1 activation.

The use of these complexes was a great advance in CO research, but there were still problems for *in vivo* studies. The requirement of a steric ligand or light to dissociate CO from the complex and the difficulty of solubilizing compounds in dimethylsulphoxide (DMSO) demanded a search for new compounds. Clark *et al.* developed tricarbonylchloro(glycinato)ruthenium(II) ([Ru9CO)3Cl (glycinate)]), also known as CORM-3, with [Mn2(CO)10] renamed as CORM-1 and [Ru(CO)3Cl2]2 as CORM-2 [151]. CORM-3 is a water-soluble compound that is able to release CO into physiological solutions without prior activation. It was shown to protect the heart from ischemia-reperfusion injury and from cardiac allograft rejection [151]. More recently, a new CORM was identified, known as sodium boranocarbonate Na2 [H3BCO2] and termed CORM-A1, which does not contain a transition metal and is water soluble. It releases CO at a slower rate when compared to others CORMS [152]. CORM-A1 was shown to have cerebroprotective effects [153,154], vasodilatory effects in the kidney [155] and antithrombotic properties [156,157]. However, it is less effective than the metal CORMs in certain aspects (e.g., bactericidal) [158]. Although several models of CORMs have been developed, the residual transition metal is potentially toxic, and further studies are required before these molecules can be applied in the clinics.

Interestingly, the use of CO as a gas is in phase I human clinical trials, and a study has been completed whereby 250 ppm of CO was inhaled by healthy volunteers (http://www.clinicaltrials.com). Other clinical trials with the use of CO inhalation are ongoing in the USA, including trials for pulmonary fibrosis, severe pulmonary hypertension and post-operative ileus following colon resection. The advances in the use of therapeutic CO reinforce the idea of using this gas in immune-dependent models, such as solid organ transplantation.

### Immunomodulatory effects of CO

In innate immunity, the induction of HO-1 increases DAF expression, which decreases complement activity and, consequently, decreases vascular injury [159]. TLR activation through IFN-β/JAK2/STAT-1/INOS/NO signaling was inhibited by the use of CORM-2, which consequently inhibited macrophage HMGB-1 release [160]. The same treatment induced tolerogenic DCs, which inhibited TLRs, maturation, pro-inflammatory cytokine secretion, proliferation of alloreactive T cells and IRF-3 expression, while maintaining IL-10 production [161]. Macrophages exposed to CO also displayed inhibition of TLR activation via impaired translocation to lipid rafts and suppressed reactive oxygen species (ROS) generation [162].

CORM-2 and CO exposure affects endothelial cell adhesion by diminishing ICAM-1 expression concurrently with reduced proinflammatory cytokine (TNF and IL-1β) production [142,163]. Other proinflammatory cytokines were affected after exposure to CO, including IL-6 and IL-17, which were downregulated in pulmonary epithelial cells through the ERK1/2 MAPK pathway [164]. This pathway inhibited by CO, also led to diminished IL-2 expression and inhibited T cell proliferation [165]. Decreases in portal venous resistance through the p38 MAPK pathway was observed when rat livers were subjected to CO [166]. This pathway was also associated with protection against oxidant-induced lung injury by CO [167].

The role of CO in NK cells is poorly understood, while another important cell of the innate immune response, neutrophils, was shown to have inhibited migration in the presence of CO [168].

Wegiel *et al.* summarized the effects of CO in different immune cells, and as previously mentioned, macrophages and DCs develop a tolerogenic phenotype upon CO treatment [169]. APCs are the major link between the innate and adaptive immune responses, and CO-treated DCs were shown to express diminished MHC class II, leading to decreased APC-induced T cell proliferation and TNF and IFN-γ production [170]. CO also inhibited the CD8^+^ T cell autoimmune response and cellular accumulation in the pancreas in diabetes model [171]. Beyond the indirect action of CO on T cells, this gas has the ability to act directly on T cells by inhibiting IL-2 production and blocking T cell proliferation [165].

**Figure 2:**
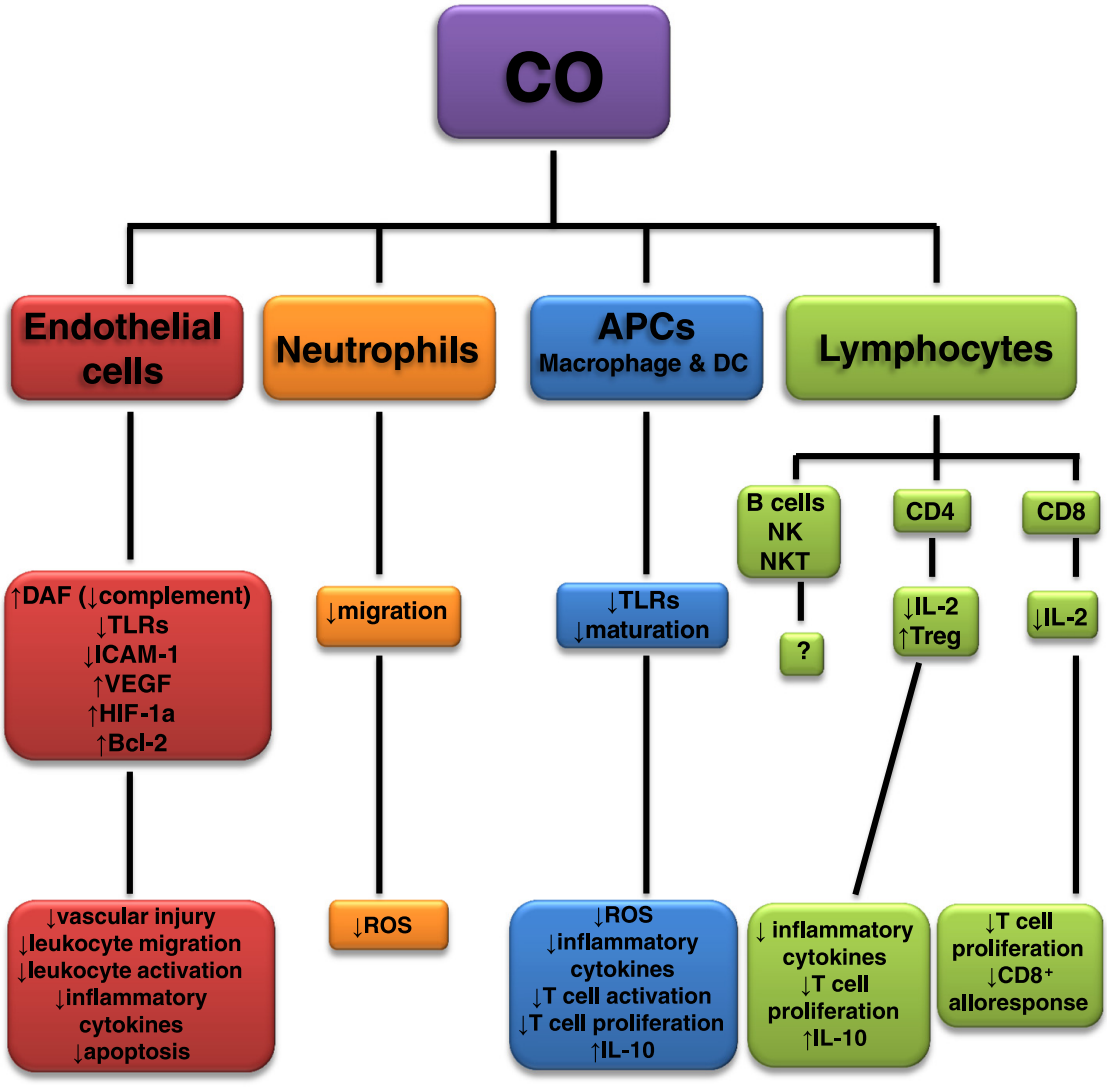
**Immunomodulatory properties of carbon monoxide (CO).** CO can act in different cells to downregulate the immune response. Endothelial cells have increased expression of decay accelerating factor (DAF), diminishing complement activation and vascular injury. These cells also have decreased Toll-like receptors (TLRs) and ICAM-1 expression in CO presence, which reduces leukocyte migration and activation, resulting in less inflammatory cytokines production. CO treatment increases vascular endothelial growth factor (VEGF), hypoxia-inducible factor (HIF)-1a and Bcl-2 expression, which is associated to apoptosis decrease. Neutrophils are also affected by CO, having impaired migration with diminished production of local reactive oxygen species (ROS). Antigen presenting cells (APCs) such as macrophages and dendritic cells (DC) have TLRs expression decreased after CO treatment, impairing their maturation leading to decreased ROS and inflammatory cytokines production, less T cell activation and proliferation and maintained IL-10 production. Although lymphocytes can be influenced by CO through APCs modulation, CO can directly act on lymphocytes by diminishing IL-2 production, which consequently suppresses T cell proliferation. CD4^+^ T cells are more prompt to develop Treg phenotype, which increases IL-10 production. CD8^+^ T cells have their alloresponse diminished when treated by CO. The role of CO on B cells, NK and NKT cells activation remains unclear.

These works corroborate the idea of using CO as an immunosuppressant during transplantation (Figure 2), which can interfere at different stages of the transplant process.

### CO in transplantation

Different organ transplant models indicated a protective role of CO administration during transplantation (Table 1).

**Table 1:** Carbon monoxide effects on organ transplantation

***ORGAN***	***FINDINGS***	***REFERENCES***
Lung	↓apoptosis, ↓inflammation, ↓oxidation, ↑tissue preservation	[194,201]
Intestine	↓inflammation, ↑graft survival, ↓apoptosis	[182,195]
Heart	↑graft survival, ↑graft function, ↑tissue preservation, ↓ischemia/reperfusion injury, ↓cell proliferation, ↓inflammation, ↓apoptosis, ↓cell infiltration, ↓cell activation, ↑Tregs	[151,173,174,181,187,189,191,198-200]
Pancreatic Islet	↑graft survival, ↓TLR4, ↓inflammation, ↓apoptosis	[172]
Liver	↑tissue preservation, ↑graft function, ↓neutrophil accumulation, ↓inflammation, ↓apoptosis	[184,186,188,196,197]
Kidney	↑graft survival, ↑graft function, ↓fibrosis, ↓ischemia/reperfusion injury, ↓apoptosis, ↓cell proliferation, ↓inflammation, ↓cell infiltration	[175-180,185,192,193,202]

↓Reduced; ↑Increased.

#### Donor

Several studies have demonstrated the relevance of HO-1/CO expression in organ donors that favor graft tolerance. In an islet allograft model, CO blocked TLR4 upregulation, diminishing the inflammatory response and cytokine-induced apoptosis, which protected the graft from rejection [172]. In a fully mismatched model, donor mice received hemin, a protoporphyrin that induces HO-1 expression, and their aortas were transplanted into non-treated mice. The neointimal area, the proliferation of endothelial cells and the production of IFN-γ by CD8^+^ T cells were reduced. The use of CORM-3 mimicked the effect of hemin, demonstrating the importance of CO in this model [173]. Donors inhaling CO or cold ischemia with CO perfusion improved graft function, and this was associated with decreased apoptosis and increased viability of endothelial cells and cardiomyocites [174]. CO has also been suggested as a potential therapy for kidney transplantation. The induction of CO in the donor by oral administration of methylene chloride was able to prevent chronic rejection of rat renal allografts [175]. Donors treated with CORM-2 presented fewer lymphocytic infiltrates and reduced acute tubular necrosis in the graft [176]. This protection was most likely related to CORM-2-induced endothelial changes via a reduction in NADPH-dependent superoxide anion production, IkB degradation, and E-selectin and ICAM-1 expression [176].

#### Graft

The use of Cobalt protoporphyrin in rapamycin-induced renal dysfunction following ischemia-reperfusion injury increased HO-1 levels and eased acute renal injury [177]. Similar results were observed with the CO inhalation model. This protection was associated with the induction of hypoxia inducible factor-1α (HIF-1α) and less severe apoptosis [178]. Cold ischemia of the liver, intestine, vein and kidney grafts in the presence of CO induced graft protection [179-184] with increased recipient survival, which was associated with increased expression of vascular endothelial growth factor (VEGF) and HIF-1α, leading to decreased apoptosis [180,181]. CO exposure during cold ischemia decreased TNF, IL-6, COX-2 and ICAM-1 expression, which led to reduced inflammation and modulated apoptosis by the increased expression of the anti-apoptotic Bcl-2 and decreased expression of the pro-apoptotic Bax through the sGC/cGMP pathway [182,184]. The use of CORMs is a promising therapy because it is a soluble method of treating organs and subjects. Kidney perfusion with CORM-3 led to improved renal function and diminished acute tubular necrosis and glomerular necrosis [176]. In warm IR, CORM-3-treated animals were protected against acute kidney injury [185]. CORM-2 prevented hepatic IR injury by elevating Bcl-2 and inhibiting caspase 3, leading to decreased apoptosis and inhibiting the proinflammatory molecules NF-kB, TNF, IL-6 and ICAM-1 [186]. Isolated heart treatment with CORM-3 showed cadioprotection and improved myocardial function [151,187]. Similar results were observed in CORM-3-treated hepatic cold preservation [188]. In a rat model, CORM-3 improved aorta graft adventitial remodeling and neo-intima formation [189]. The combination of CO and biliverdin treatment for heart and kidney grafts resulted in protection against ischemia-reperfusion injury [190].

#### Recipient

Abdominal aortic transplants presented prolonged survival with CORM-2 treatment in a murine allograft model [191]. Kidney graft recipients exposed to CO after surgery displayed improved graft function and diminished ischemia-reperfusion injury [192]. In a chronic allograft nephropathy model, inhaled CO improved renal function with decreased tubular atrophy and decreased fibrosis. Impaired anti-donor IgG antibodies and decreased expression of macrophage inflammatory protein 1 (MIP-1a), chemokine receptors (CCR1, CXCR3, CXCR5), ICAM-1 and IL-2, leading to reduced T cell proliferation, were also observed [193]. In a lung transplant model, recipient animals were exposed to CO following surgery. As a result, a marked reduction in apoptosis, inflammation and tissue damage was observed in CO-subjected mice [194]. CO administration during small intestinal transplantation also reduced inflammation, with decreased levels of IL-6, IL-1β, iNOS and COX-2 in the graft and prolonged graft survival [182,195]. Recipients treated with CO gas presented improved graft function in a liver transplantation model due to the inhibition of proinflammatory molecules, such as TNF, ICAM-1 and iNOS, leading to decreased neutrophil accumulation and diminished necrosis [196]. Similar results were obtained when recipient rats were treated by methylene chloride in a liver transplant model; the recipients displayed increased survival, impaired CD95/FasL-mediated apoptosis and preserved hepatic architecture and function [197].

In murine heart xenotransplantation, the transplanted heart with inhibited HO-1 was rapidly rejected from the recipient rat in comparison with the wild-type graft, suggesting the importance of HO-1 production by the graft. Nevertheless, treatment of both the donor and the recipient with CO prolonged the graft survival independently of HO-1 inhibition by blocking platelet aggregation and endothelial cell apoptosis [198]. Allogeneic transplanted aortic segments develop arteriosclerotic lesions. CO exposure was able to inhibit the hyperplasia associated with chronic graft rejection with fewer graft infiltrating macrophages, CD3^+^, CD4^+^ and CD8^+^ T cells. The macrophages were also less activated and presented diminished MHC class II and ICAM-1 expression. These effects were dependent on guanylate cyclase activation and cGMP generation via activation of the p38/MAPK pathway and expression of the cell cycle inhibitor p21^clip1^[199]. In a heart allograft model, the combination of HO-1, CO and bilirubin treatments led to long-term survival and tolerance of the graft by inducing Foxp3+ Tregs [200]. In a rodent model of lung transplantation from deceased donors, the combination of CO gas and biliverdin treatment induced cytoprotection by attenuating MPO, IL-8 and TNF in the graft and by oxidation, with low levels of malonaldhyde and superoxide dismutase [201].

More recently, Hanto *et al.* introduced the use of a device that can deliver CO by mg/kg, which is an advance for future therapeutic CO administration. They showed reduced DGF in a kidney allograft swine model [202].

Interventions with CO were efficient at different points of the transplant (Table 2). Summarizing these studies, CO appears to play an important role in controlling the immune response and graft acceptance. However, further investigation is required concerning the phenotypes of cells (DC, macrophages, T cells) after CO treatment during transplantation and to confirm the described tolerogenic effect of CO in different models. It would also be interesting to further analyze the dose of CORM and the resulting side-effects prior to starting use in humans. Nevertheless, CO is a good candidate for potential changes in the clinical setting.

**Table 2:** Carbon monoxide immunomodulation during transplantation

***CO TARGET***	***CONSEQUENCES***
DONOR	↓Toll-like receptor (TLR)4
	↓endothelial cell proliferation
	↓lymphocytic infiltration
	↓inflammatory cytokines production (IFN-g)
	↓apoptosis
	↓Reactive oxygen species (ROS)
	↓NFκB (IκB degradation)
	↓E-selectin/ ICAM-1
GRAFT	↑Hypoxia inducible factor (HIF)-1a
	↑Vascular endothelial growth factor (VEGF)
	↓apoptosis (↑Bcl-2, ↓Bax, ↓caspase 3)
	↓inflammatory cytokines production (TNF, IL-6)
	↓prostaglandin (COX2)
	↓ICAM-1
	↓NFκB
RECIPIENT	↓Ischemia and reperfusion injury
	↓fibrosis
	↓anti-donor IgG antibodies
	↓chemokine receptors (CCR1, CXCR3, CXCR5)
	↓chemokines (IL-8, MIP-1a)
	↓ICAM-1
	↓IL-2 (↓T cell proliferation)
	↓leukocyte infiltration (CD3+, CD4+, CD8+ T cells and macrophages)
	↓macrophage activation (↓MHC class II)
	↓neutrophil activation (↓MPO)
	↓apoptosis (↓CD95/FasL)
	↓inflammatory cytokines production (IL-1β, TNF)
	↓iNOS
	↓prostaglandin (COX2)
	↓platelet aggregation
	↑cell cycle inhibition (↑p21clip1)
	↑Treg (Foxp3+ T cells)

## Conclusions

Our current knowledge about CO completely disrupts the idea that it is only a dangerous gas. Instead, it shows that we are capable of manipulating it and can strategically use it for clinical purposes. In this review, we highlighted the protective properties of CO associated with its capacity to modulate the immune system. CO was shown to downregulate components and cells of the innate immune response, thereby impairing inflammation and the activation of the adaptive immune response. Moreover, CO was able to directly act on adaptive immune cells, which play a primary role in allograft rejection. Due to its capacity to immunomodulate the environment, this intervention was effective during the three stages of transplantation (donor, graft and recipient), widening the possibilities of its use. In conclusion, CO has the capacity to downmodulate the immune response, suggesting its use as an attractive therapeutic agent during transplantation.

## Abbreviations

CO: Carbon monoxide;CORMs: Carbon monoxide releasing molecules;DGF: Delayed graft function;HO-1: Heme oxygenase-1;MAPK: Mitogen activated protein kinase;NO: Nitric oxide;IR: Ischemia and reperfusion

## Competing interests

The authors have no competing interests.

## Authors' contributions

MA wrote the manuscript; NC wrote and corrected the manuscript. Both authors read and approved the final manuscript.

## Authors' information

MA – Postdoctoral research fellow at the Laboratory of Transplantation Immunobiology, Institute of Biomedical Sciences, University of Sao Paulo, Brazil.

NC – Professor and Head of the Department of Immunology, Institute of Biomedical Sciences, University of Sao Paulo, Brazil.
